# Social Network Analysis as a Methodological Approach to Explore Health Systems: A Case Study Exploring Support among Senior Managers/Executives in a Hospital Network

**DOI:** 10.3390/ijerph15030511

**Published:** 2018-03-13

**Authors:** Aoife De Brún, Eilish McAuliffe

**Affiliations:** School of Nursing, Midwifery and Health Systems, University College Dublin, Dublin 4, Ireland; eilish.mcauliffe@ucd.ie

**Keywords:** social network analysis, leadership, organizational research, health, management

## Abstract

Health systems research recognizes the complexity of healthcare, and the interacting and interdependent nature of components of a health system. To better understand such systems, innovative methods are required to depict and analyze their structures. This paper describes social network analysis as a methodology to depict, diagnose, and evaluate health systems and networks therein. Social network analysis is a set of techniques to map, measure, and analyze social relationships between people, teams, and organizations. Through use of a case study exploring support relationships among senior managers in a newly established hospital group, this paper illustrates some of the commonly used network- and node-level metrics in social network analysis, and demonstrates the value of these maps and metrics to understand systems. Network analysis offers a valuable approach to health systems and services researchers as it offers a means to depict activity relevant to network questions of interest, to identify opinion leaders, influencers, clusters in the network, and those individuals serving as bridgers across clusters. The strengths and limitations inherent in the method are discussed, and the applications of social network analysis in health services research are explored.

## 1. Introduction

Health systems research is a growing field, which utilizes multiple methods and perspectives in understanding and improving how systems operate. An important milestones for health systems research was the World Health Organization (WHO)’s *World Health Report 2012: No Health Without Research* [1]. The World Health Organization has defined a health system as “all the organizations, institutions and resources that are devoted to producing health actions” and improving health the objective of any health system [2]. Health systems research studies the organization of people, institutions, and resources that deliver health care services to meet the health needs of target populations. A health systems perspective recognizes the inherent complexity of healthcare, the interdependent nature of components within the system, and the challenges of disentangling and studying component parts of the system. To fully comprehend and depict the complexities of such dynamic and open systems, new and innovative methods from other fields of research are required to describe and evaluate systems. Social network analysis is one set of methodologies that offers a different research lens and enables the mapping of complex systems. This paper will describe how the method can be employed to understand, explore, chart changes over time, and assess the impact of interventions in health systems.

Social network analysis (SNA) represents a distinctive set of methodologies to map, measure, and analyze social relationships between people, teams, and organizations [3]. It facilitates the exploration of patterns and types of relationship between actors, where these actors (which may be individuals, groups or organizations) are visually represented in a network map by structural nodes, and relationships (ties or links) between these nodes. Nodes may also be used to represent events, ideas, or actions, depending on the focus of the research. SNA allows for the analysis of the role and influence of actors in a network; it can also characterize and map network relationships, as well as analyze the structure of a system [3]. SNA provides a theoretical approach to explore the interaction of actors in a system and a framework to test theories around collective behavior and social interaction [4]. Using mathematical tools and specialized software packages [5], researchers can explore how patterns of relationships can operate to facilitate or inhibit communications, actions, and capacities in systems. SNA has applications in many fields, including health behavior, public health and prevention, and organizational and group research [6]. It has been employed in a myriad of healthcare settings [7], though often as a descriptive rather than an evaluative tool.

The social network approach involves identifying, or asking participants to identify, key members of their network in relation to a question of interest. Responses to network questions may be binary, indicating the presence of a tie or relationship, or value-based, emphasizing the strength of that tie or relationship. For instance, the sample network question “Does your team rely on *Person A* for leadership?” could be coded as yes (1) or no (0), whereas a question asking “To what extent does your team rely on *Person A* for leadership?” could be coded on a Likert-type scale, in order to more clearly differentiate between leadership strength in the network.

A network can be composed of three components: meaning, direction, and weight. Meaning refers to the network question(s) of interest—for example, leadership behavior or information sharing. Direction refers to the type of link, as links can be undirected or directed towards specific individuals. Furthermore, directed links can be one-way (provides information to Person A) or two-way (has a bi-directional information sharing relationship with Person A). Finally, relationships can also be weighted or valued. For instance, the network question may ask individuals to report their frequency of information-sharing on a scale from ‘never’ to ‘daily’, and this can be used to assess the strength or frequency of information-sharing in a network.

In order to begin planning a social network study, relevant individuals or organizations in a system can be identified using various methods, including document reviews and interviews with stakeholders. A robust method for generating self-reported ties is to use recall lists [8], where a list of all individuals or organizations is provided to participants, and respondents are asked to indicate their relations to others named on the roster. This enables the collection of whole network data, and the researchers clearly define the network, meaning this is a “bounded” or defined approach to the definition of the network. In contrast, *not* using a roster of names asks people to report the names of those relevant to the network question of interest, but this approach may be vulnerable to recall bias and omissions. This is called the “egocentric” approach. Rich network data can be generated using myriad data collection approaches. Observations, interviewing, surveys, and archive record reviews may be employed to assess ties of interest among members of a group [4]. Although challenging to achieve, completeness of data is important in SNA, as the method is more sensitive to missing data than other research methods. A network response rate of 75% is typically required in SNA for data to be considered reliable [9]. This can be challenging to achieve in longitudinal studies.

By collating network data, different types of information and insights can be gleaned from the resulting network metrics and maps. Firstly, the general structure of a network, in terms of its cohesion and shape, are two properties that can be informative. Cohesion refers to the number of connections within a network; the more connections, the higher the density of the network. The network shape relates to overall distribution of ties in the network, and can be useful to differentiate the core actors (those that are highly connected and prominent) in a network from those on the periphery (those with loose or few links) [10,11].

Whilst depiction of the network map offers a useful overall view of the network structure, additional metrics may be computed to further interrogate network characteristics, in order to illuminate how the network operates, and who may be more or less influential in the network in various ways. These metrics include centrality, degree, betweenness, and centralization (see Table 1 for a summary of SNA metrics and definitions). Although many more metrics exist and can be employed for various purposes, these are among the most commonly used metrics, and will be employed here as a means of illustrating how social network analysis can provide various insights into understanding the operation of a healthcare network or health system.

Although SNA has often been used in healthcare settings for descriptive purposes, it has less frequently been employed in the evaluation of networks change following intervention, and there are no established standards for the evaluation of networks [14]. Yet, previous research has confirmed the positive impacts of networks when they operate well. Effective networks can increase efficiency in information flow and diffusion, and can facilitate access to information, expertise, and innovation [7,15,16,17]. Cunningham et al. [15] found that cohesive and collaborative professional networks in healthcare can enable the effective coordination of care and enhance the quality of care delivered.

SNA is especially useful in leadership research, as it can create awareness of others in the network, identify gaps, and can operate as a means of bolstering connections and strengthening the capacity of the network to act collectively [12]. Furthermore, network perspectives view leadership as embedded in the relationships connecting people, and therefore SNA, as a highly suitable method for mapping and analyzing these relationships [14,18]. Despite the applicability of SNA to leadership research, relatively little empirical work has been conducted on leadership in social networks [14,18,19]. This paper employs a case study approach to illustrate the value of social network methods in understanding health systems. The case study draws on research that has analyzed the leadership network among a group of senior hospital managers/executives in a newly-formed hospital group, to explore if collective approaches to leadership developed as a result of eleven hospitals being placed into a networked structure. This paper highlights the value of social network approaches in health systems research, using our case study as an illustration.

## 2. Materials and Methods

### 2.1. Context

This study examined the senior leadership network in one hospital group in Ireland. Hospital groups were established relatively recently, with the remit of encouraging collaborative working across organizational boundaries and enhancing integration of care and services for a defined geographical population group [20]. The hospital group in this study serves a population of 1.1 million in the eastern part of Ireland. It is anticipated that the network or group will encourage learning across the hospitals in the group and will result in standardization and improvements in access to quality care. The results presented in this paper are part of a larger study and research programme [21], and the data reported herein are used to highlight the value of SNA in health systems research, and illustrate the types of useful and practical information that may be drawn from the resulting analysis.

### 2.2. Participants

Participants were eligible to take part in the study if they were members of the senior management team of the hospital group or one of the 11 hospitals within the group. The hospital group management team, as well as the Chief Executive Officer or General Manager, Director of Nursing, and Clinical Director of each of the 11 hospitals in the hospital group formed the initial cohort for this work (*n* = 44).

### 2.3. Measures

A social network survey was designed where the members of the identified cohort responded to questions addressing the frequency of their contact and collaboration with other members of the network, as well as their sources of formal or informal support within the network. This illustrative case study will focus on one network question posited regarding the support relationships in the network. The roster method was not used for this question, to avoid unduly overburdening participants. Instead, an open-ended question was used to encourage participants to report those they contacted for any form of support in the previous month.

### 2.4. Data Collection and Procedure

As the purpose of the main study was to analyze the development of the network over time, the study design involved the collection of data at six monthly intervals. The same data was collected at each time point. This paper focuses only on the data collected at time points 1 and 2, as our purpose here is to illustrate the potential of SNA methods, rather than describe the development of the network over the period of the study.

Eligible participants were invited to take part following a presentation from the research team. Each person was emailed and asked to provide their consent to take part in the study. Participants received a link to complete the survey online. The network survey was hosted online by *Qualtrics* (www.qualtrics.com), and the link was emailed to relevant individuals who had consented to take part in the study. Two reminders were issued to participants, two and three weeks after the initial link was circulated at each time point. As new members joined the network, they received an email invite from the research team to participate. The first round of network data was collected in September and October 2016, and the second round occurred in April 2017.

### 2.5. Data Analysis

The social network data enabled the generation of a network map and computed network metrics, including centrality, centralization, density, distance, bonding, and bridging, to understand how the system was operating at each of the three time points. Survey data were analyzed using descriptive statistics and entered into UCINET (version 6.619) [5] and Netdraw (version 2.159) [22], software to generate network maps and to compute relevant network metrics [4].

### 2.6. Ethical Considerations

Favorable ethical opinion for this study was obtained from University College Dublin’s Research Ethics Committee (LS-16-20).

## 3. Results

### 3.1. Participation

Of the 44 individuals eligible to take part at time point 1, one person opted out, and 35 took part, representing an 81% response rate. Typical of new networks, there was considerable growth of the network in the six months between time points 1 and 2, with some turnover in roles. At time point 2, 52 individuals were eligible to take part and 33 participated, representing a lower response rate of 63%. Additional participants represented further roles within the hospital group management team.

### 3.2. Network Maps

**Figure 1:**
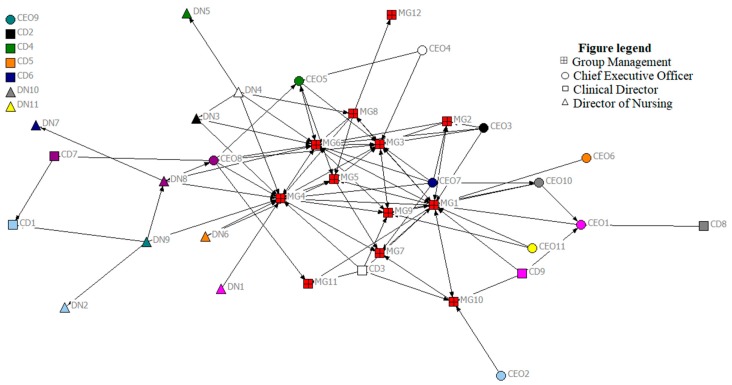
Support relationships across the network—time point 1 (nodes at top left are those with no reported network ties).

**Figure 2:**
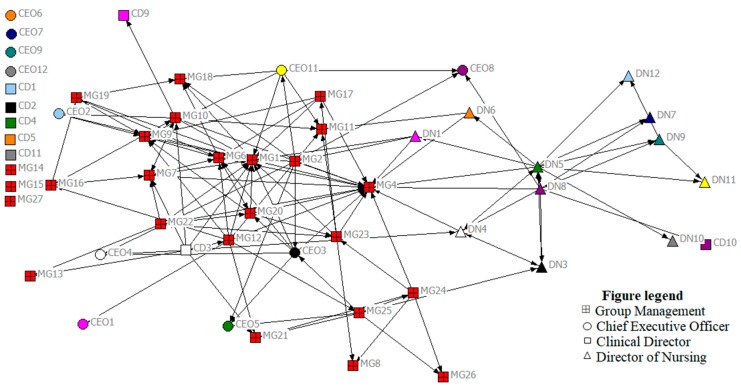
Support relationships across the network—time point 2 (nodes at top left are those with no reported network ties).

Figure 1 and Figure 2 display the network maps, generated using Netdraw, and indicating support relationships reported in the network at time points 1 and 2, respectively. Various colors on the map represent different places of work (either the hospital group executive, i.e., the overarching governance team for the entire hospital group (red), or one of the eleven hospitals in the group). Various shapes represent the different roles of individuals in the network: Chief Executive Officers/General Managers (CEO), Clinical Directors (CD), Directors of Nursing (DN), and members of the group management team (MG). Network and node-level metrics, computed using UCINet, are displayed in Table 2 and Table 3. Colors represent the same sites in both network maps.

### 3.3. Network-Level Metrics

Network-level metrics provide information about how the network as a whole is operating and assess the degree to which the network is focused on one or a few nodes (in this study, nodes represent individuals in the network). These results communicate information about the entire network, including its structure and degree of cohesion. Network-level indicators analyzed below include density, core-periphery structure, and centralization (Table 2).

#### 3.3.1. Density

From the resulting map at time point 1, overall, we can see that the number of ties is low. Network metrics indicate a density score of 0.06 (or 6%) at time point 1 and 5% at time point 2. This confirms that only a small fraction of possible support relationships currently exist in the network. This is perhaps to be expected, given that people may only look to individuals whom they trust (something which takes time to develop) in a network for support-seeking information; communicating about other issues may not require the same level of trust, and is therefore likely to happen with a larger number of colleagues. Given the rate of change and growth in the network between time points 1 and 2, the continuity of interaction required to build trusting relationships is difficult, so it is not surprising to see a slight drop in the density of support relationships. However, the average number of links per node has increased across the time points, from 2.28 to 2.45. This highlights the need to explore both the metrics of density and number of links per node, to fully understand changes across time when there is change in the size of the network.

#### 3.3.2. Centralization

We can also discern that support relationships are quite decentralized, in that there is not a high density of relationships at the center of the map. Instead, these relationships are spread out across the network map. At the network level, the centralization metric provides an overall indicator of how clustered ties are to one or a few individuals in the network, where scores closer to 1 indicate a highly centralized network, and figures closer to 0 indicate a decentralized network. In the current study, the centralization value was 0.19 at time point 1 and 0.16 at time point 2, indicating a relatively decentralized network. A decentralized network may be more resilient, as the network is not dependent on only a few individuals, and instead social influence and ties are more evenly distributed, and tend to be less hierarchical [16]. Therefore, if one or two central individuals were removed or left the network, it would not leave a major gap in support for individuals, i.e., the decentralized nature of relationships can act as a protective factor, and ensure continuing support is available for most in the network. The impact is likely to be more significant if one or two central individuals were removed from a highly centralized and hierarchical network.

#### 3.3.3. Network Structure: Core-Periphery

Network structure or shape relates to distribution of ties across the entire network. It can be useful to differentiate the core actors—that is, those that are highly connected in a network from those on the periphery (those with fewer ties). Although the network is not highly centralized, it is evident from the maps at both time points that there is a cluster of red towards the center of the map, indicating that individuals on the hospital group executive team are quite often the source of support for others in the network. Given that the hospital group had only recently been established, others in the network are relying on this group as a primary source of support. This core–periphery structure is confirmed through network metrics, which report that seven individuals acted as the core of the network at Time 1 (MG1, MG3, MG4, MG5, MG6, MG8, and MG9). By Time 2 however, this group of core actors in the network has expanded to included 16 people (CEO3, CD3, DN5, DN8, MG1, MG2, MG4, MG6, MG7, MG10, MG9, MG12, MG20, MG22, MG23, and MG25), indicating that more individuals are now central to the support network in the group, compared to six months previously.

### 3.4. Node-Level Metrics

Whilst network-level metrics provide information about the network as a whole, node-level metrics provide useful information about individuals in the network, and can identify who in the network is more or less influential regarding the network question of interest. In this section, we demonstrate some of these key node-level metrics, including degree, betweenness and bridging (Table 3).

#### 3.4.1. Degree

Degree represents the number of links to a person (*in-degree*) and from a person (*out-degree*). Degree metrics are often used as a way to identify visible people or opinion leaders in a network. As the network question of interest here relates to support in the network, we were interested in knowing who the principal sources of support were in the network. Therefore, particularly important and influential were those people who were highest in “in-degree”, i.e., those who were approached for support. Interestingly, it is evident from the network metrics that those most contacted for support changed over time, but that two individuals (MG1 and MG4) were amongst the most contacted at both time points. Although these individuals were frequent sources of support, the decentralized nature of the support relationships means that the network did not overly rely on these individuals. This effectively demonstrates the importance of measurement at multiple time points to capture the dynamic nature of networks.

#### 3.4.2. Betweenness and Bridging

Betweenness is especially relevant to leadership research, as it reveals the frequency a person lies on the shortest path connecting everyone else in the network, i.e., those individuals that can most easily connect other network members. Those highest in betweenness are said to occupy strategic positions in the network, and can serve as bridgers to others in the network [23]. In the current study, MG1 and MG6 were highest in betweenness at both time points, but by the next time point, DN5 was highest-scoring on betweenness. While we know who the important actors are on this dimension, we do not know why these individuals have become important. For this reason, it is important to use network maps to generate discussion among participants, and reflect on the actions or events that may have resulted in these changes. Qualitative research is often an important part of social network analysis, to aid interpretation of network maps. Close bonding between members of a sub-group in a network may indicate close working relationships or a sense of trust between those in a particular cluster or “clique”. Bridgers are those individuals high in betweenness that serve to link individuals or clusters to other parts of the network. They may be identified as important gatekeepers in the network, and can be effective in diffusing information across the network.

## 4. Discussion

This paper has described the value of social network analysis as a methodology to explore, depict, and understand the operation of networks and systems. This case study, assessing the support relationships between senior leaders in a recently established hospital network, illustrated some of the principal network- and node-level metrics used in social network analysis, and demonstrates the value of these maps and metrics to understand the system. SNA offers a valuable approach to health systems and services researchers, as it offers a means of depicting activity relevant to network questions of interest to identify opinion leaders, influencers, or clusters in the network, and those individuals serving as bridgers in the network.

At the network level, the results revealed a very low density of support relationships, which is perhaps not surprising given the relatively recent establishment of the hospital group. Interestingly, the support relationships were dispersed across the network, indicating that the network is decentralized in this respect, and not overly dependent on one or a few individuals. This may act as a protective mechanism for the network going forward, in that if further turnover and change was observed, it would not adversely impact the support systems available within the network. Furthermore, given that this is a new network, as people get to know each other through enhanced collaboration, we may expect to see a growing number of support relationships emerge over time. This hypothesis highlights the value of measuring social networks at multiple time points. Social networks are dynamic and constantly evolving. Taking a snapshot of networks over time can reveal important changes, developments, and opportunities, or equally may highlight vulnerable areas of the network. Multiple time measurements can also illustrate the effects of interventions targeting development in the network, and can help to capture the dynamic nature of networks which are adapting to change.

At the individual node level, this method illuminated the “core” or central actors in the network; in this case, those who were contacted most for support in the network. Using metrics such as betweenness and in-degree, we could identify those that occupied strategic positions in the network, and those that were most visible and influential. Importantly, SNA can account for different kinds of centrality; actors in a network may be influential, but the approach enables understanding of the *type* of role they play in their central position. For example, betweenness is an especially useful metric for leadership studies, as it measures the degree to which a node occupies a strategic position in a network [6], and has been described as a strong predictor of perceived leadership [24,25]. Similarly, degree can identify opinion leaders in a network, or act as a measure of social integration in the network. SNA also allows for the identification of those acting as bonders or bridges in a network, serving to connect parts of the network that may otherwise be isolated. These individuals, acting as gatekeepers, play an important role in diffusion across the network [13]. Knowledge of the links and bridgers in the network can be employed strategically to addresses gaps in the network and build on strengths.

While this method offers a visual representation of collected data, researchers are cautioned against misinterpretation and the limitations and risks inherent in the method. Hoppe and Reinholt [12] highlight four of the risks of SNA that must be considered carefully in the design and analysis stages: a lack of privacy and related ethical/transparency issues, drawing conclusion from incomplete data, oversimplification of analysis and misreading data, and incorrect interpretation of network measures. Given these risks, it is advised that interpretation occur with participants using the resulting network maps for discussion purposes, and allowing members of the network to explain what they feel is occurring and how well the network is functioning [12]. Social network analysis offers a holistic view of a network, when often only parts of that network are familiar to actors. Balkundi and Kilduff contend that an accurate perception of a network can itself be a source of power in an organization, and can better enable leaders to develop collaborations [14]. This is particularly important in health systems, where frequent reconfigurations occur and can sometimes result in skepticism and a reluctance to engage (i.e., a “wait and see” attitude). Additionally, network metrics can be misleading; for instance, density is one metric which may present interpretation challenges. According to Valente [26], greater network density does not necessarily mean the network is more efficient. Too much density may create redundancy, and reduce the ability of those in the network to access outside sources of information and influence, a situation which may leave the network vulnerable to groupthink. Therefore, metrics such as density should be interpreted with caution.

It is important to recognize another limitation of the method: the influence of those beyond the network. Bounded or unbounded network studies are unlikely to capture the full extent and influence of all relevant actors. The influence of network actors is likely to be affected by ties that may not show up at all in the organizational charts [14]. This is a limitation of the current study and inherent in research of this nature. There is a need to achieve balance between the scope, and therefore the number of individuals to include in a social network study, and the potential response burden on participants, especially in studies employing the roster method (where all individuals are listed in the network question).

SNA may be employed for descriptive or evaluative purposes, and it is often employed as a diagnostic tool, as it can identify opinion leaders (and potential change agents) as well as highlighting intervention points, by pinpointing weaknesses or gaps in the network [7]. Similarly, SNA can be used to understand what parts of the network are working well, and this can be used to target qualitative research to understand the contextual conditions and mechanisms that are enabling effective network operation. Social network analysis is growing in popularity, and the software and analytical techniques are constantly evolving. The value of social network analysis for leadership research [14,18] and more broadly, health systems and organizational research, has long been touted [3,6,25]; however, the technique has not yet received widespread use. For instance, it is not often applied to health systems research in low- and middle-income countries [3]. With this paper, we have aimed to describe the approach and provide examples of the possible methods of data collection, as well as the information that may be extracted from social network data in helping to explore, depict and understand health systems.

## 5. Conclusions

This case study described the results of a study exploring support among senior managers/executives in a newly established hospital group. Network analysis offers a valuable approach to health systems, and services researchers as it offers a means of depicting activity relevant to network questions of interest, identifying opinion leaders, and those individuals serving as bridgers in the network. We have highlighted the value of the methodology as a tool to depict, diagnose, and analyze networks in health systems, and the approach can also be employed to evaluate interventions aimed at enhancing the network’s operation. According to a scoping review of social network analysis in healthcare [7], the potential of the method in health research is considerable, yet it is only relatively recently that researchers have begun to employ the method in healthcare settings. We hope this case study highlights the benefits and strengths of the approach in enabling insight into health system networks.

## Figures and Tables

**Table 1:** Summary of relevant network and node measures.

Measure	Description
*Centralization*	Network-level measure that can assess the degree to which network links are focused on one or a few nodes in the network [6]. A centralized network is indicated when there is a high standard deviation of centrality scores, because some individuals have high centrality and some have low centrality. Conversely, a network is said to be decentralized when the standard deviation from centrality scores is low, as everyone in the network has similar centrality scores.
*Core-Periphery*	Core–periphery structures are network where there is a densely connected group of nodes and others who are more loosely connected.
*Density*	Density explores the number of ties in a network as divided by the number of potential ties. Links per node is also recommended for use alongside density to guard against the potential of misinterpretation of density score [12].
*Degree centrality*	Degree is an often-used measure which examines the number of links to a person (in-degree) and from a person (out-degree). Valente [6] describes degree as a useful measure to identify opinion leaders in a network and/or as a measure of social integration.
*Betweenness centrality*	The frequency a person lies on the shortest path connecting everyone else in the network. This metric is valuable, as it indicates the degree to which a node occupies a strategic position in a network). Betweenness centrality is indicative of bridging, which refers to those individuals in a network who function to serve as the link diverse others. For instance, bridging may indicate access to new resources or links to external groups; having “bridgers” in a network is important in terms of linking to otherwise disconnected or distant groups and may indicate access to new resources. It is calculated using betweenness centrality [13].

**Table 2:** Network-level metrics for support.

Network metrics	Time 1	Time 2	Change
Density	0.06	0.05	−00.1
Distance-based cohesion	0.12	0.1	−0.02
Average number of links per person	2.28	2.45	+0.17
Degree centralization	0.19	0.16	−0.03
Average distance	2.14	2.95	+0.81
Diameter (max distance) (SD)	5 (0.94)	9 (1.65)	+4

**Table 3:** Node-level metrics for support.

Node Metrics	Time 1	Time 2
Highest in-degree (person contacted most for support)	MG 4 (14)	MG1 (14)
	MG6 (13)	MG4 (17)
	MG1 and MG3 (12)	MG9 (8)
Greatest number of links	MG1 (22 ties)	MG1 (19 ties)
	MG4 (19 ties)	MG4 (17 ties)
	MG6 (18 ties)	MG6 (15 ties)
Highest in betweenness	MG1 (154)	DN5 (163)
	MG 6 (69)	MG1 (124)
	MG 4 (48)	MG6 (123)
